# Photobiomodulation in the aging brain: a systematic review from animal models to humans

**DOI:** 10.1007/s11357-024-01231-y

**Published:** 2024-06-11

**Authors:** Lucía Rodríguez-Fernández, Candela Zorzo, Jorge L. Arias

**Affiliations:** 1 Neuroscience Laboratory, Department of Psychology, University of Oviedo, Oviedo, Spain; 2 INEUROPA, Instituto de Neurociencias del Principado de Asturias, Oviedo, Spain; 3 ISPA, Instituto de Investigación Sanitaria del Principado de Asturias, Oviedo, Spain

**Keywords:** Photobiomodulation, Aging, Brain, Cognition, Emotion

## Abstract

Aging is a multifactorial biological process that may be associated with cognitive decline. Photobiomodulation (PBM) is a non-pharmacological therapy that shows promising results in the treatment or prevention of age-related cognitive impairments. The aim of this review is to compile the preclinical and clinical evidence of the effect of PBM during aging in healthy and pathological conditions, including behavioral analysis and neuropsychological assessment, as well as brain-related modifications. 37 studies were identified by searching in PubMed, Scopus, and PsycInfo databases. Most studies use wavelengths of 800, 810, or 1064 nm but intensity and days of application were highly variable. In animal studies, it has been shown improvements in spatial memory, episodic-like memory, social memory, while different results have been found in recognition memory. Locomotor activity improved in Parkinson disease models. In healthy aged humans, it has been outlined improvements in working memory, cognitive inhibition, and lexical/semantic access, while general cognition was mainly enhanced on Alzheimer disease or mild cognitive impairment. Anxiety assessment is scarce and shows mixed results. As for brain activity, results outline promising effects of PBM in reversing metabolic alterations and enhancing mitochondrial function, as evidenced by restored CCO activity and ATP levels. Additionally, PBM demonstrated neuroprotective, anti-inflammatory, immunomodulatory and hemodynamic effects. The findings suggest that PBM holds promise as a non-invasive intervention for enhancing cognitive function, and in the modulation of brain functional reorganization. It is necessary to develop standardized protocols for the correct, beneficial, and homogeneous use of PBM.

## Introduction

Aging is a multifactorial biological process that may be associated with physical and cognitive decline [1] and an increase in susceptibility to neurodegenerative diseases, such as Alzheimer’s Disease (AD) or Parkinson’s Disease (PD) [2].

The World Health Organization (WHO) estimates that by 2030, 1 in 6 people will be in their sixties and beyond, increasing from 1 billion in 2020 to 1.4 billion. Also, people aged 80 and older are expected to triple between 2020 and 2050 [3]. This increased longevity and life expectancy may be a result of multiple protective factors, such as an improvement of the healthcare system and sanitary conditions, nutrition, or psychological factors, among others [4]. However, with regard to increased longevity, it is important to consider the quality of life during aging: many older people will be affected by age-associated cognitive decline, the main cause of disability [1].

In normal aging, there is a deterioration of cognitive processes such as attention, learning, verbal fluency, and reaction time [5]. The severity and engagement of alterations in other cognitive functions, especially memory and executive function, are observed in mild cognitive impairment (MCI) and dementia [6]. In addition, older adults can suffer from affective mental disorders, including anxiety and depression [7].

It is known that during aging, the function of the nervous system deteriorates over time (senescence), which may result in age-related disorders, such as a higher susceptibility to infectious diseases, autoimmune and degenerative processes, or cancer [8]. Regarding age-related cellular and molecular changes, a reactive immune phenotype has been shown to develop, with an up-regulation of pro-inflammatory cytokines [9]. Aging is linked to the exhaustion of the regenerative capacities of the nervous system, including reduced adult hippocampal neurogenesis, plasticity decrease, demyelinating conditions, brain hypoperfusion, and blood–brain barrier dysfunction [10, 11]. Moreover, it has been pointed out that aging is associated with mitochondrial dysfunction, which is present in both normal aging and aged-related disorders. Thus, mitochondrial dysfunction can be caused by alterations in mitochondrial autophagy—that is, mitophagy—, resulting in alterations in the respiratory capacity, reducing the mitochondrial membrane potential [12–15].

Altogether, this highlights the importance of applying strategies to prevent or delay decline [16], with non-pharmacological interventions gaining increased attention. Photobiomodulation (PBM) is a non-pharmacological therapy that has a promising application to treat diverse neurological illnesses linked to age, such as AD and PD [17]. PBM involves the utilization of red or near-infrared (NIR) light with low power density to stimulate, preserve and regenerate cells and tissues. The procedure entails placing one or multiple light sources on the head with the aim of stimulating a specific cerebral area. The emitted radiation may originate from a laser or a light emitting diode and can be used in either pulsed or continuous modes [18]. It has been observed that PBM increases cerebral blood flow and energy metabolism in the brain, and it also has antioxidant effects [19]. Also, one of the most accepted theories about the effects of PBM postulates that it causes activation of the mitochondrial enzyme cytochrome c oxidase (CCO), leading to an increase in the production of mitochondrial adenosine triphosphate (ATP), which, in turn, may improve the cell’s metabolic activity [20]. This is possible due to the capacity of mitochondrial acceptors to absorb photonic energy (for a review, see [21]). Likewise, PBM favors the expression of genes associated with tissue regeneration and repair [20]. The effects of PBM have also been observed in active brain networks, showing a modulation of brain metabolic activity, and leading to greater metabolic efficiency in healthy rodents [22].

Given the accumulating evidence supporting the beneficial effects of PBM, experimental studies are increasingly concentrating on its application in the context of aging, a field of knowledge that is growing faster. Thus, this systematic review aimed to compile the preclinical and clinical evidence of the effect of PBM during aging in both healthy and pathological conditions. This includes behavioral analysis and neuropsychological assessment, as well as brain-related modifications.

## Method

The present systematic review followed the Preferred Reporting Items for Systematic reviews and Meta-Analyses (PRISMA) statement, and the Joanna Briggs Institute (JBI) critical appraisal tools [23, 24]. Concerning methodology and methodological quality, we ranked the papers based on their reliability and credibility, following the JBI appraisal tools. The selection of the studies included scientific articles whose main topic was the intervention during the aging process using the PBM, excluding the reviews and case reports. Papers lacking results or with unsupported results were promptly excluded from the review. In instances of dependency, articles were assessed according to the questions outlined by the JBI. All articles were assessed with the critical appraisal tool for randomized studies by the JBI.

### Search strategy

The search was conducted in PubMed, Scopus, and PsycInfo databases on July 25, 2023, and updated on April 1st, 2024. Articles included in this review were restricted to those published from 2003 to the present.

PubMed MeSH database was used to define the keywords that were used as a search index. A total of 23 keywords were selected and combined: (ageing) OR (aging) OR (elderly) OR (old people) OR (old rat) OR (old mice) AND (photobiomodulation) OR (low level light therapy) OR (low level laser therapy) AND (brain) OR (cognit*) OR (memor*) OR (learn*) OR (executive function) OR (emotion) OR (anxiety) OR (depression) OR (Alzheimer’s disease) OR (Parkinson’s disease) OR (neurodegeneration) OR (mild cognitive impairment) OR (dementia) NOT (review).

### Selection criteria

Articles were limited to the following inclusion criteria: (a) studies examining the effect of PBM; (b) aging; (c) human studies; (d) rodent studies; (e) assessment of brain activity (f) assessment of cognitive function; (g) assessment of emotional processes. Articles that omitted the inclusion criteria and/or met the following exclusion criteria were not included: (a) reviews, case reports, case studies, conference papers, correspondence, editorials, letters to the editor, editor’s notes, other editorial materials, and commentaries; (b) articles with no experimental results; (c) articles with only descriptive data.

### Study selection

**Fig. 1:**
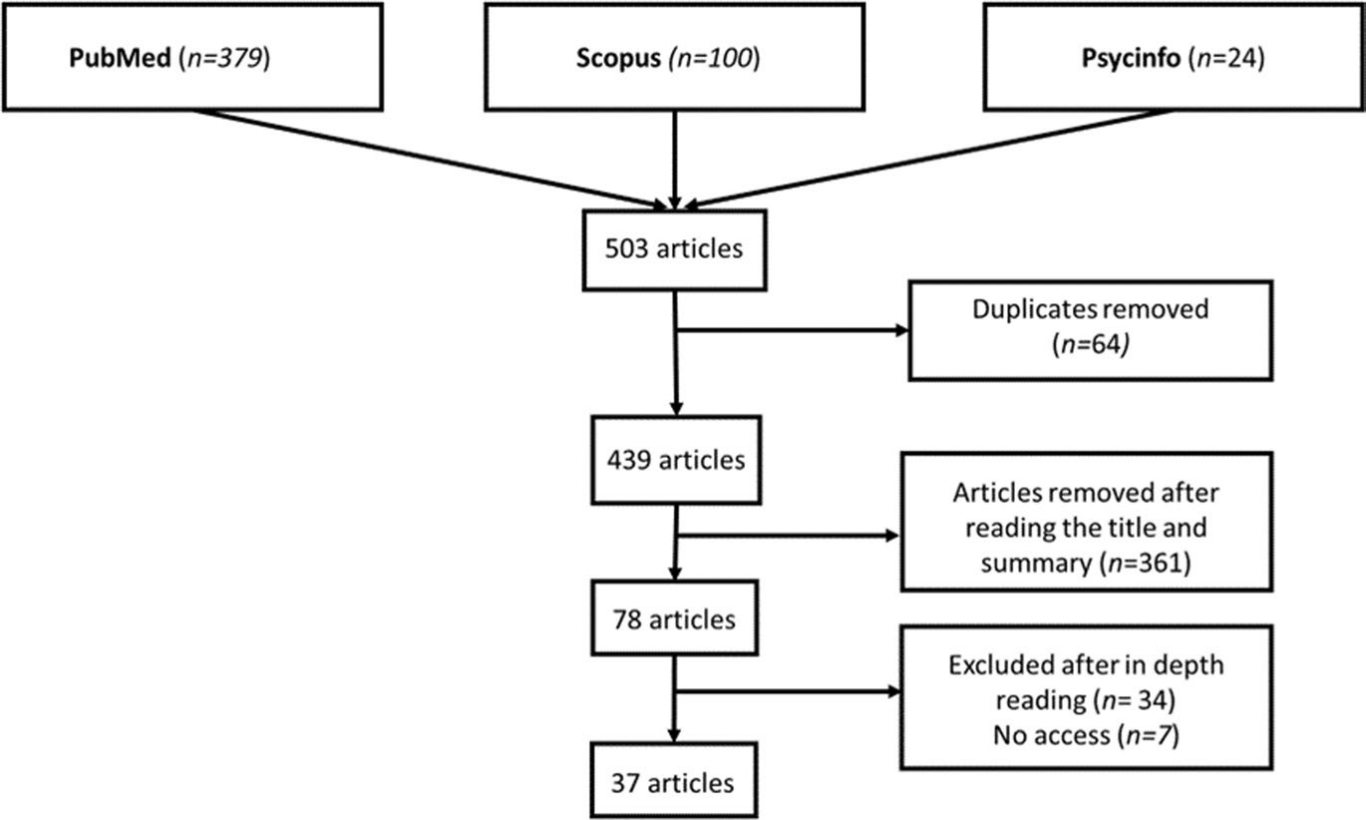
Literature flow diagram of the selection process in the different phases of the systematic review

Firstly, searches were carried out in the PubMed, Scopus, and Psycinfo databases, collecting 379, 100, and 24 articles, respectively, leading to 503 potential articles. Second, articles duplicated across databases were removed through Mendeley and manually, resulting in 361 articles analyzed by 2 authors independently (LRF, CZ). Then, the title and abstract of each article were carefully read to discard those that did not match the inclusion criteria, obtaining a total of 78 articles, and finally, the articles were read, discarding those that did not match the inclusion criteria or could not be retrieved, leaving a total of 37 articles. All these procedures were (Fig. 1). Two independent investigators (LRF, CZ) performed the search. One of them (LRF) removed duplicate articles, while both investigators screened the titles, abstracts, and full texts of the articles, and assessed them according to the inclusion and exclusion criteria (LRF, CZ).

### Data analysis

The selected articles were exhaustively analyzed, and their main methods and results were added to Table 1 (for the animal models) and Table 2 (for the human studies).

**Table 1:** PBM^1^ application on animal sample

*Authors*	*Animal (sex, age, model)*	*Total sample size / groups (n)*	*Behavioral method*	*Behavioral results*	*Brain results*	*PBM parameters (Wavelength nm / Wavelength type (frequency)/ intensity/ irradiance)*	*Brain target area*	*Days / Anesthesia*
*Buendía *et al*. (2022)*	Mouse (male, 9 months, AD^2^ model)	N = 23 / Control (n = 5); Control PBM (n = 7); AD control (n = 4); AD PBM (n = 7)	Recognition memory (Novel Object Recognition test) Locomotor activity (Open field)	PBM did not improve recognition memory in an AD model PBM did not alter locomotor activity in an AD model	PBM rescued fEPSP^3^, LTP^4^ and partially restored LTD^5^ in an AD model, while no differences were found in PPF^6^	630 nm/ NR^7^/ NR/ 1 W	Generalized in a box	5 consecutive days/ No anesthesia
*Cardoso *et al*. (2021) a*	Rat (male, 4 and 20 months, healthy)	N = 30/ Young control (n = 8); Young PBM (n = 7); Aged control (n = 7); Aged PBM (n = 8)	–	–	PBM reversed high concentration of several metabolic pathways in aged rats in the cortex (aspartate, glutamate, ammonia recycling, urea cycle, purine, arginine, proline, alanine, phenylalanine, tyrosine, glutathione metabolism, phosphatidylcholine biosynthesis, glycine and serine) PBM increased concentration of acetate and guanosive tryphosphate in aged rats in the hippocampus	810 nm/ Continuos /15 J/ 100 mW	5 irradiation points (coordinates: 1 = AP + 4.20 mm and ML 0.00 m; 2 = AP –5.52 mm and ML + 6.60 MM; 3 = AP –3.00 mm and ML + 6.60 mm; 4 = AP 0.00 mm and ML 0.00 mm; 5 = AP -5.52 mm and ML 0.00 mm)	58 consecutive days/ No anesthesia
*Cardoso *et al*. (2021) b*	Rat (male, 4 and 20 months, healthy)	N = 64/ Young control (n = 15); Young PBM (n = 15); Aged control (n = 16); Aged PBM (n = 18)	Spatial memory (Barnes maze). Associative memory (Inhibitory Avoidance). Anxiety (Elevated Plus Maze). Locomotor activity (Open field test)	PBM improved spatial memory in aged rats No differences were found in associative memory, anxiety, and locomotor activity	PBM reduced the cortical expression of IL-5^8^ and increased IL-6^9^, IL–10^10^, and TNF-α^11^ in aged rats PBM reduced the hippocampal expression of IP-10^12^ and fractalkine in aged rats	810 nm/ Continuous / 15 J/ 100 mW	5 irradiation points (coordinates: 1 = AP + 4.20 mm and ML 0.00 m; 2 = AP –5.52 mm and ML + 6.60 MM; 3 = AP –3.00 mm and ML + 6.60 mm; 4 = AP 0.00 mm and ML 0.00 mm; 5 = AP –5.52 mm and ML 0.00 mm)	58 consecutive days/ No anesthesia
*Cardoso *et al*. (2021) c*	Rat (male, 20 months, healthy)	N = 11 / Aged control (n = 5); Aged PBM (n = 6)	–	–	PBM increased the cortical expression of STAT3^13^, ERK^14^, and JNK^15^, while no differences were found in Akt^16^, p70S6K^17^, STAT5^18^, and p38, in aged rats PBM increased the hippocampal expression of p70S6K, STAT3 and Akt, while no differences in STAT5, ERK, JNK and p38	810 nm/ Continuous / 15 J/ 100 mW	5 irradiation points (coordinates: 1 = AP + 4.20 mm and ML 0.00 m; 2 = AP –5.52 mm and ML + 6.60 MM; 3 = AP –3.00 mm and ML + 6.60 mm; 4 = AP 0.00 mm and ML 0.00 mm; 5 = AP –5.52 mm and ML 0.00 mm)	58 consecutive days/ No anesthesia
*Cardoso *et al. *(2022) a*	Rat (male, 3 and 20 months, healthy)	N = 24/ Young control (n = 7); Young PBM (n = 5); Aged control (n = 6); Aged PBM (n = 6)	–	–	PBM increased the CCO^19^ activity in the ventral basal thalamic nucleus–lateral, caudal caudate–putamen, and nucleus of cranial nerve 3, CA1 of the hippocampus, mammillothalamic tract, auditory cortex, and primary and secondary visual cortex in aged rats PBM reduced the CCO activity of anterodorsal thalamic nucleus in aged rats	810 nm/ Continuous / 15 J/ 100 mW	5 irradiation points (coordinates: 1 = AP + 4.20 mm and ML 0.00 m; 2 = AP -5.52 mm and ML + 6.60 MM; 3 = AP –3.00 mm and ML + 6.60 mm; 4 = AP 0.00 mm and ML 0.00 mm; 5 = AP –5.52 mm and ML 0.00 mm)	58 consecutive days/ No anesthesia
*Cardoso *et al.,* (2022) b*	Rat (male, 20 months, healthy)	N = 10/ Aged control (n = 5); Aged PBM (n = 5)	–	–	PBM reduced the cortical expression of ERK and p38 and increased the activation of STAT3 and ERK PBM increased the hippocampal expression of p70S6K and STAT5 and reduced the expression of p38 PBM decreased cortical levels of IL–5, and hippocampal levels of IL–5, IL–18^20^ and fractalkine levels, while increments in IL–1α^21^ both in the hippocampus and cortex	660 nm/ Continuous / 15 J/ 100 mW	5 irradiation points (coordinates: 1 = AP + 4.20 mm and ML 0.00 m; 2 = AP –5.52 mm and ML + 6.60 MM; 3 = AP –3.00 mm and ML + 6.60 mm; 4 = AP 0.00 mm and ML 0.00 mm; 5 = AP –5.52 mm and ML 0.00 mm)	10 consecutive days/ No anesthesia
*Cho *et al*. (2018)*	Mouse (male, early intervention started at 2 months and delayed intervention started at 6 months of age, AD model)	NR/ AD PBM early intervention (n = 8–12); AD PBM delayed intervention (n = 8–12); AD control (n = 8–12)	Spatial memory (MWM^22^). Associative memory (Passive avoidance test). Anxiety (Elevated plus maze test)	PBM improved spatial memory in an AD model, and reduced anxiety phenotype No differences were found in associative memory	PBM with early intervention showed a reduction in Aβ plaques, microgliosis, and increased degradation of Aβ proteins and insulin–degrading enzyme in the cortex of an AD model, while no differences were found with delayed interventions, or in the hippocampus	610 nm/ NR/ 2 J/cm^2^ / 1.7 mW	2 irradiation points (midpoint of the parietal bone and the posterior midline of the seventh cervical vertebra)	48 non–consecutive days (3 times per week)/ Anesthesia
*De Taboada *et al*. (2011)*	Mouse (male, 3 until 9 months, AD model)	N = 100/ AD control (n = 20); AD continuous PBM (n = 20); AD pulsed PBM 40 mW (n = 20); AD pulsed PBM 200 mW (n = 20); AD pulsed PBM 400 mW (n = 20)	Spatial memory (MWM)	PBM continuous and pulsed (40, 200, and 400 mW) improved spatial memory in an AD model	PBM reduced Aβ plaques. While there was an increase of sAβPPα protein levels Only pulsed wave at 400 mW and 200 mW decreases amyloid load in the cerebrospinal fluid PBM with NIR leads to a reduction in inflammatory markers, a restored of ATP^23^ levels and an increase in c-fos protein expression in AD model	808 nm/ Continuous and pulsed (100 Hz)/ NR/ 40 mW, 200 mW, 400 mW	1 irradiation point (midline of the skull, 4 mm caudal to the coronal suture)	72 non-consecutive days (3 times per week)/ Anesthesia
*El Massri *et al*. (2018)*	Mouse (male, 3 and 12 months, healthy)	N = 16/ Young control (n = 5); Aged control (n = 6); Aged PBM (n = 5)	-	-	PBM reduces astrocytes and microglia in the striatum. No differences were found in pavalbumin and encephalopsin interneurons, nor in striatal dopaminergic terminals	670 nm/ NR/ NR/ NR	NR	8 consecutive months/ NR
*Grillo *et al*. (2013)*	Mouse (female, tested at 3, 7, and 12 months, AD model)	NR / AD control (n = 3–4); AD PBM (n = 3–4)	–	–	PBM reduced APP^24^, β–amyloid, and alpha–b-c8rystalin in the hippocampus in AD model PBM upregulated members of the heat shock protein signaling pathways in AD model	1072 nm/ Pulsed (600 Hz)/ NR/ 5 mW	6 irradiation points (enclosing the animals, irradiating in all directions from all 6 sides)	40 non–consecutive days (2 times per week)/ No Anesthesia
*Hosseini *et al*. (2022)*	Mouse (male, NR, an aging–induced protocol)	N = 50 / Control (n = 10); Aged control (n = 10); Aged PBM 8 J (n = 10); Aged PBM 16 J (n = 10) Aged PBM 32 J (n = 10)	Spatial memory (Lashley III maze). Asociative memory (Passive avoidance test). Sociability (Three–chamber social interaction test)	PBM with 8 and 16 J/cm^2^ improved spatial memory in aging–induced mice, and social memory with 8 J/cm^2^ No differences were found in associative memory, and social interaction, or with 32 J/cm^2^	PBM with 8 and 16 J/cm^2^ increased GAP–43^25^ and SYN^26^ expression in the hippocampus in aging-induced mice, while no effects were found in PSD–95^27^, or with 32 J/cm^2^ PBM with 8 and 16 J/cm^2^ reduced IL–6 hippocampal expression and 8 J/cm^2^ reduced TNF– α	810 nm/ Pulsed (10 Hz)/ 8, 16 and 32 J/ 4.75 W/cm^2^	1 irradiation point (approximately bregma)	24 non–consecutive days (3 times per week)/ No Anesthesia
*Li *et al*. (2023)*	Mouse (male, 4 until 6 months, AD model)	NR/ AD control (n = 3–10); AD PBM 16 J/cm^2^ (n = 3–10); AD PBM 32 J/cm^2^ (n = 3–10)	Spatial memory memory (MWM) Recognition memory (Novel object recognition test)	PBM with 32 J/cm^2^ improved spatial memory and memory recognition in an AD model, but not with 16 J/cm^2^	PBM significantly reduced density of Aβ plaque and the reduction was similar between prefrontal cortex and hippocampus PBM mitigated Aβ burden in the brain by improving lymphatic clearance of Aβ and increased diameter of the basal MLVs^28^	1267 nm/ NR/ 16 and 32 J/cm^2^ / NR	1 irradiation point (sagittal sinus region)	7 non–consecutive days (once every two days)/ Anesthesia
*Lutfy *et al*. (2024) a*	Rats (male, 2 months and 16 months, SD^29^)	N = 48/ Young control (n = 8); Aged control (n = 8); Young SD (n = 8); Aged SD (n = 8); Young SD PBM (n = 8); Aged SD PBM (n = 8)	Short–term memory (Y–Maze) Anxiety (Elevated plus maze test)	PBM did not improved short-term memory in a SD aged model PBM reduced anxiety phenotype	PBM up–regulated Bcl-2 and BDNF^30^ in the hippocampus in aged SD, reversed Ach^31^, AchE^32^, MAD^33^, and SOD^34^ expression, while no differences were found in GSH^35^ or hippocampal histoarchitecture	830 nm/ Continuous / 71.96 J/ 100 mW	6 irradiation points (sagittal on each side of the longitudinal commissure and between bregma and lambda)	3 consecutive days/ No anesthesia
*Lutfy *et al*. (2024) b*	Rats (male, 2 months and 16 months, SD.)	N = 48/ Young control (n = 8); Old control (n = 8); Young SD (n = 8); Old SD (n = 8); Young SD PBM (n = 8); Old SD PBM (n = 8)	–	-	PBM increased hypothalamic CCO activity, BDNF and Bax in aged SD, reversed GSH, SOD activity, and histological features, reduced TNF–α, IL–6 and CRP^36^, while no differences were found in MDA^37^	830 nm/ Continuous / 71.96 J/ 100 mW	6 irradiation points (on two sides of the animal head)	3 consecutive days/ No anesthesia
*Mohammed *et al*. (2023)*	Rats (male, NR, PD^38^ model)	N = 21/ Control (n = 7); PD control (n = 7); PD PBM (n = 7)	Locomotor activity (Open field)	PBM restored locomotor activity	PBM decreased MDA, NO^39^ and GSH for midbrain and striatum PBM recovered AchE, and monoaminoxidase enzymatic activity in midbrain, while no effects were found in Na + , K + –ATPase. PBM restored 5–HT^40^ and NE^41^ levels in the midbrain and striatum while no significant differences were found in DA^42^	830 nm/ Continuous / 71.96 J/ 100 mW	6 irradiation points (three on each side of the longitudinal commissure and between bregma and lambda)	14 consecutive days/ No anesthesia
*Oueslati *et al*. (2015)*	Rat (female, NR, PD model)	First evaluation: N = 35/ Control (n = 5); PBM 5 mW/cm^2^ (n = 6); PBM 10 mW/cm^2^ (n = 6); PBM 20 mW/cm^2^ (n = 6); PBM 25 mW/cm^2^ (n = 6); PBM 30 mW/cm^2^ (n = 6) Second evaluation: N = 23/ PBM 5 mW/cm^2^ (n = 7) and a PBM 2.5 mW/cm^2^ (n = 7); Control (n = 9)	Locomotor activity (Cylinder test)	PBM with 2.5, 5 and 10 mW/cm^2^ improved locomotor activity (akinesia)	PBM groups showed less nigral dopaminergic degeneration, with a significant protection against α–syn-induced toxicity only on the higher fluence group as well as less striatal fiber denervation with a significant effect observed after treatment at higher fluence. No differences were found in cell survival or cortical cell density	808 nm/ NR/ NR/ 2.5, 5, 10, 15, 20, 25, 30 mW/cm^2^	Above the head	14 consecutive days + 6 weeks treatment withdrawal + 8 consecutive days/ No anesthesia
*Salehpour *et al*. (2017)*	Mouse (male, NR, artificially aging-induced protocol)	N = 72/ Control (n = 12); Aged control (n = 12); Aged PBM 660 nm 4 J/cm^2^ (n = 12); Aged PBM 810 nm 4 J/cm^2^ (n = 12); Aged PBM 660 nm 8 J/cm^2^ (n = 12); Aged PBM 810 nm 8 J/cm^2^ (n = 12)	Spatial memory (Barnes maze) Episodic memory (What-where-which task)	PBM with red and NIR and 8 J/cm^2^ improved spatial memory and episodic-like memory in aging induced mice No differences were found with 4 J/cm^2^	Red and NIR PBM with 8 J/cm^2^ augmented active mitochondria, MMP^43^, ATP, and CCO activity while no differences were found with 4 J/cm^2^ Red and NIR PBM with 4 and 8 J/cm^2^ reduced ROS^44^ production There was a significant reduction of Bax to Bcl–2 ratio, and caspase-3 with 8 J/cm^2^ Red and NIR 8 J/cm^2^ lasers notably drop caspase11357_2024_12313 protein levels	660 and 810/ Pulsed (10 Hz)/ 4 and 8 J/cm^2^/ 200 mW	Above the head	18 non–consecutive days (3 times per week)/ No Anesthesia
*Salehpour *et al*. (2018)*	Mouse (male, 2 months and 18 months, healthy)	N = 45 / Young control (n = 15); Aged control (n = 15); Aged PBM (n = 15)	Spatial memory (Barnes Maze) Locomotor activity (Open field test)	PBM improved spatial memory in aged rats No differences were found in locomotor activity	PBM increased ATP in the hippocampus	660 nm/ Continuous/ 16 J/cm^2^/ 200 mW	1 irradiation point (3 mm rostral to a line drawn through the anterior base of the ears)	14 consecutive days/ No anesthesia
*Shen *et al*. (2020)*	Mouse (NR, NR, AD model)	NR / AD control (n = 4–14); AD PBM (n = 4–14)	Spatial memory (Y-maze and MWM)	PBM improved learning and memory in an AD model	PBM reduced Aβ depositions in an AD model in the cortex and hippocampus PBM reduced p-JNK and c-Jun signals around plaques PBM increased MKP7^45^ phosphorylation while inhibited the phosphorylation of JNK3^46^ and PSD-95 and AMPA receptor endocytosis PBM rescued the decrease of dendritic spines in an AD model. PBM increased synaptophysin and MAP-2^47^ in the cortex and hippocampus of an AD model	635 nm/ NR/ 6 J/cm^2^/ 8.75 mW	Above the head	30 consecutive days/ No Anesthesia
*Sipion *et al*. (2023)*	Mouse (male and female, 1 until 6 months, AD model)	N = 60/ Control (n = 19); PBM 5 mW (n = 20); PBM 470 mW (n = 21)	Spatial memory (MWM) Short-term spatial memory (Y maze) Recognition memory (Novel object recognition test)	No differences were found in spatial memory, short-term spatial memory, and memory recognition	PBM did not altered the numbers of neurons in the prefrontal cortex or the amyloid plaque load PBM had no effect regardless of intensity use	810 nm/ Pulsed (100 Hz)/ NR/ 5 mW and 470 mW	Above the head	40 non-consecutive days (2 times per week)/ No anesthesia
*Tao *et al*. (2021)*	Mouse (female, 4 and 12 months AD model)	N = 40–48 / Control (n = 10–12); AD control (n = 10–12); AD PBM 10 Hz (n = 10–12); AD PBM 40 Hz (n = 10–12)	Spatial memory (MWM) Recognition memory (Novel object recognition test)	PBM with 10 Hz improved spatial memory and recognition memory in an AD model, but not with 40 Hz	PBM with 10 and 40 Hz reduced the number of Aβ plaques in the hippocampus and cortex in an AD model of 12 months PBM with 10 Hz increased the colocalization between microglia and Aβ in the cortex of mice, while no differences were found with astrocytes, and it reduced M1-like microglia	1070 nm/ Pulsed (10 and 40 Hz)/ 4.5 J/cm^2^/ 25 mW/cm^2^	Generalized in a box	60 consecutive days/ No anesthesia
*Xu *et al*. (2024)*	Mouse (NR, 4, 8 and 12 months, AD model)	N = 40/ 4 months control (n = 5); 4 months continuous PBM 808 nm (n = 5); 4 months pulsed 40 Hz PBM 808 nm (n = 5); 4 months pulsed 40 Hz visible light (n = 5); 8 months control (n = 5); 8 months continuous PBM 808 nm (n = 5); 8 months pulsed 40 Hz PBM 808 nm (n = 5); 8 months pulsed 40 Hz visible light (n = 5); 12 months control (n = 5); 12 months continuous PBM 808 nm (n = 5); 12 months pulsed 40 Hz PBM 808 nm (n = 5); 12 months pulsed 40 Hz visible light (n = 5)	Spatial memory (MWM)	PBM improved spatial memory with 808 nm (continuous and 40 Hz), and visible light	PBM reduced Aβ plaques in the old mice but not on the young mice. Plaques amount seemed to be least for mice treated by 808 nm and visible LED in 40 Hz PW^48^ mode Cerebral amyloid angiopathy was not found in AD model mice from 808 nm 40 Hz group in vivo	808 or visible / Continuous and pulsed (40 Hz)/ NR/ 50 mW/cm^2^	Generalized in a box	7 consecutive days/ No anesthesia
*Zhang *et al*. (2024)*	Mouse (male, 18–20 months, PND^49^)	NR/ Control (n = 6–10); PND (n = 6–10); PND PBM 80 J/cm^2^ (n = 6–10); PND PBM 160 J/cm^2^ (n = 6–10)	Short and long-term spatial memory (Barnes maze). Locomotor activity (Open field test)	PBM with 80 and 160 J/cm^2^ improved short and long-term spatial memory, and memory recognition in a PND model. No differences were found in locomotor activity	PBM decreased ROS and TFN- α PBM upregulated the expression of IRF7^50^, reduce microglia M1 and increase M2 phenotype, upregulated the expression of BDNF, CCO and improved ATP production restoring enzyme activity of complexes I, II, IV It altered the profiles of mRNA in the prefrontal cortex and hippocampus and reversed expression of inflammasome proteins	810 nm/ Continuous /80 and 160 J/cm^2^/ 80 mW/cm^2^	Above the head	5 consecutive days/ No anesthesia

1.**PBM** = photobiomodulation. 2. **AD** = Alzheimer’s disease. 3. **fEPSP** = excitatory field potentials. 4. **LTP** = long-term potentiation. 5. **LTD** = long-term depression. 6. **PPF** = paired-pulse facilitation. 7.**NR** = not reported. 8. **IL-5** = interleukin 5. 9. **IL-6** = interleukin 6. 10. **IL-10** = interleukin 10. 11. **TNF-α** = tumor necrosis factor alpha. 12. **IP-10** = interferon gamma-induced protein. 10. 13. **STAT3** = signal Transducer and Activator of Transcription 3. 14. **ERK** = extracellular-Signal-Regulated Kinase. 15. **JNK** = c-Jun N-terminal kinases. 16. **Akt** = protein kinase B. 17. **p70S6K** = ribosomal protein S6 kinase beta-1. 18. **STAT5** = signal Transducer and Activator of Transcription 5. 19**. CCO** = cytochrome C oxidase. 20. **IL-18** = interleukin 18. 21. **1L-1α** = interleukin 1 alpha. 22. **MWM** = morris Water Maze. 23. **ATP** = adenosine triphosphate. 24. **APP** = amyloid precursor protein**.** 25. **GAP-43** = growth-associated protein-43. 26. **SYP** = synaptophysin. 27. **PSD-95 = **post-synaptic density-95. 28. **MLV** = meningeal lymphatic vessels. 29. **SD** = sleep deprived. 30. **BDNF** = brain-derived neurotrophic factor. 31. **Ach** = acetylcholine. 32. **AchE** = Acetylcholinesterase. 33. **MAD** = malondialdehyde. 34. **SOD** = super oxide dismutase. 35. **GSH** = glutathione. 36. **CRP** = c-reactive-protein. 37. **MDA** = measured malondialdehyde. 38. **PD** = Parkinson’s disease. 39. **NO** = nitric oxide. 40. **5-HT** = serotonine. 41. **NE** = norepinephrine. 42. **DA** = dopamine. 43. **MMP** = mitochondrial membrane potencial. 44. **ROS** = reactive oxygen species. 45. **MKP7** = Mitogen-activated protein kinase phosphatase 7. 46. **JNK3** = c-Jun N-terminal Kinase 3. 47. **MAP2** = microtubule associated protein-2. 48. **PW** = pulsed wave. 49.**PND** = postoperative neurocognitive disorder. 50. **IRF7** = interferon regulatory factor 7

**Table 2:** PBM^1^ application on human sample

*Authors*	*Age (Gender, model)*	*Total sample size / groups (n)*		*Neuropsychological assessment*	*Neuropsychological results*	*Brain results*	*PBM parameters (Wavelength nm / Wavelength type (frequency)/ intensity/irradiance)*	*Target*	*Duration*
*Arakelyan* (2005)	Mean: 73.1 years (37% men, AD ^2^)	N = 145/ AD control (n = 15); AD PBM (n = 25); AD MFT^3^ (n = 25); AD LCT^4^ (n = 17); AD LMLCT^5^ (n = 25); AD Pharmacotherapy (n = 38)		Cognitive function (ADAS-cog^6^)	PBM improved cognition in AD	-	633 nm/NR^7^/ NR /4 mW	Intravenous application	6 days over 18 months
*Bullock *et al*. (2021)*	Mean: 67.7 years (60% men, PD^8^)	N = 20/ PD PBM protocol 1 (n = 10); PD PBM protocol 2 (n = 10)		Cognitive function (MoCA^9^)	PBM did not affect general cognition in PD	-	904 nm/ Pulsed (50 Hz)/ 42 J/ 60 mW/diode	Transcranial application with 4 irradiation points (lateral cranial point, midline cranial point, parietal region point, and intranasal)	Protocol 1: 1 month placebo (3 times per week) + 1 month washout + 1 month PBM/placebo (2 and 1 times per week, respectively) Protocol 2: 1 month PBM (3 times per week) + 1 month washout + 1 month PBM/placebo (1 and 2 times per week, respectively)
*Chan *et al*. (2019)*	Range: > 60 years (10% men, healthy)	N = 30/ Aged control (n = 15); Aged PBM (n = 15)		Global cognitive function (CDRS^10^) Verbal learning (HKLLT^11^) Depressive symptoms (CGDS^12^) Anxiety symptoms (BAI^13^)	PBM leads to a positive effect on cognitive inhibition and lexical/semantic access in healthy aging PBM leads to a faster reaction time and better fluency (generating total words) in healthy aging PBM did not result in depressive and anxiety symptoms amelioration	-	633 nm and 870 nm/ Continuos / NR/ 999 mW	Transcranial application with 3 irradiation points (right and left frontopolar regions [FP1^14^, FP2^15^, and Pz^16^])	One day
*Chan *et al*. (2021)*	Mean: 66.3 years (50% men, MCI^17^)	N = 18/ MCI control (n = 9); MCI PBM (n = 9)		Visual memory (Computerized Corsi block test)	PBM leads to improvements in visual memory in MCI	PBM reduced frontal lobe HbO^18^ in easier and difficult levels of a visual memory task, in MCI	810 nm/ Continuous / 7 J/cm^2^/ 20 mW/cm^2^	Transcranial application with 9 irradiation points in the frontal lobe (placed to F7^19^, AF7^20^, Fp1, FpZ^21^, Fp2, AFZ^22^ and FZ^23^)	One day
*Chao (2019)*	Mean: 79.8 ± 5.8 years (37.5% men, AD or dementia)	N = 8/ AD control (n = 4); AD PBM (n = 4)		Cognitive function (ADAS-cog; NPI^24^)	PBM improved cognition and neuropsychiatric symptomatology in AD or dementia	PBM increased cerebral perfusion and connectivity between posterior cingulate cortex and lateral parietal nodes in AD or dementia, increased CBF^25^ in parietal cortex, and did not alter the default-mode-network	810 nm/ Pulsed (40 Hz)/ 720 J/ 100 mW	Transcranial application with 2 irradiation points (posterior and anterior) and intranasal application	36 non-consecutive days (3 times per week)
*Fear *et al*. (2023)*	Mean: 68 years (40% men, healthy)	N = 7 / Aged Pre and Post PBM		-	-	PBM increased ATP^26^ synthase flux	670 nm/ NR/ NR/ NR	Transcranial application with 1 irradiation point (occipital lobes)	4 consecutive days
*Hu *et al*. (2023)*	Mean: 64.74 ± 5.73 years (16% men, healthy)	N = 61/ Aged Pre and Post PBM		Working memory (N-back task)	PBM (single and repeated) improved memory on day 1 and day 7, on healthy ageing. These results were maintained during the follow up at day 14, 21 and 28	PBM (single) decreased HbO activity during the memory task in the right hemisphere, and PBM (repeated) decreased HbO activity in both hemispheres	1064 nm/ NR/ 3.4 J/ 250 mW/cm^2^	Transcranial application with 1 irradiation point (left DLPFC^27^)	7 consecutive days
*Lee & Chan. (2023)*	Range: 50–80 years (43% men, healthy)	N = 30/ Aged Pre and Post PBM		Visual memory (computerized visual span task)	PBM did not improved visual memory	PBM reduced HbO in a difficult level of visual memory task, but not in the easier level	810 nm/ Continuous/ 7 J/cm^2^/ 20 mW/cm^2^	Transcranial application with 9 irradiation points (F7, AF7, Fp1, Fpz, Fp2, AF8, F8, Fz and Cz)	One day
*Liebert *et al*. (2021)*	Range: 60–80 years (41% men, PD)	N = 12/ PD PBM Protocol 1 (n = 6); PD PBM Protocol 2 (n = 6)		Cognitive function (MoCA)	PBM improved the cognitive function with both protocols	-	Transcranial: 810 nm/ Pulsed (40 Hz) / 240 J/ 200 mW/cm^2^ Intranasal: NR/ NR/ 15 J/ NR Transdermal and Transabdominal: 904/ Pulsed (50 Hz)/ 3.6 J/ 47 mW/cm^2^	Transcranial, intranasal, transdermal and transabdominal	Transcranial: Protocol 1: 144 non-consecutive days (3 times per week for 4 weeks + 2 times per week for 4 weeks + 1 time per week for 4 weeks + 3 per week for 40 weeks). Protocol 2: 99 non-consecutive days (3 times per week for 4 weeks + 2 times per week for 4 weeks + 1 time per week for 4 weeks + 3 per week for 24 weeks) Intranasal: NR Transdermal and transabdominal: Same protocol as transcranial
*Nagy *et al*. (2021)*	Range: 65–75 years (50% men, AD)	N = 60/ AD control (n = 30); AD PBM (n = 30)		Cognitive function (MoCA)	PBM improved the cognitive function in AD	-	650 nm/ NR/ NR/ NR	Intranasal application	36 non-consecutive days (3 times per week)
*Papi *et al*. (2022)*	Mean: 64.13 ± 4.73 years (0% men, MCI)	N = 42/ MCI control (n = 21); MCI PBM (n = 21)		Cognitive function (MMSE^28^) Attention (Go/No-Go task)	PBM increased the cognitive function, and specifically attention	-	850 nm/ NR/ 60 J/ 400 mW	Transcranial application with 1 irradiation point (Fp2)	One day
*Qu *et al*. (2022)*	Mean: 65.6 ± 5.41 years (20% men, healthy)	N = 86/ Aged control (n = 25); Aged PBM (n = 61)		Working memory (N-back tasks)	PBM improved working memory in healthy ageing. There was a higher accuracy in two of the testing conditions and lower response time on the more difficult condition	-	1064 nm/ Continuos / 120 J/ 250 mW/cm^2^	Transcranial application with 1 irradiation point (left DLPFC)	7 consecutive days
*Razzaghi *et al*. (2024)*	Mean: 74.66 ± 14.4 in PBM groups, and 75.85 ± 7.19 years in control groups (77% men, AD and MCI)	N = 13/ AD and MCI control (n = 7); AD and MCI PBM (n = 6)		Cognitive function (MoCA) Anxiety symptoms (HAM-A^29^) Depression symptoms (HDRS^30^)	PBM improved the cognitive assessment in AD and MCI Both groups significantly improved depression levels, but not anxiety levels	-	810 nm/ Pulsed (40 Hz)/ 300 J/ 150 mW/cm^2^	Transcranial application with 3 irradiation points (frontal, occipital and temporal lobe)	72 non-consecutive days (6 times per week)
*Saucedo *et al*. (2021)*	Range: 56–85 (23% men healthy)	N = 68/ Aged control (n = 33); Aged PBM (n = 35)		-	-	PBM increased oxidized CCO^31^ in right and left prefrontal cortex in healthy ageing, decreased HbR in right prefrontal cortex, and did not modified prefrontal HbO	1064 nm/ Continuos / 120 J/cm^2^/ 250 mW/cm^2^	Transcranial application with 1 irradiation point (right anterior prefrontal cortex [P2])	One day

1**. PBM** = photobiomodulation. 2. **AD** = Alzheimer’s disease. 3. **MFT** = magnetic field therapy. 4. **LCT** = light chromotherapy**.** 5**. LMLCT** = combined PBM, MFT and LCT. 6. **ADAS-cog** = Alzheimer’s disease assessment Scale- Cognitive subscale. 7. **NR** = not reported. 8. **PD** = Parkinson’s disease. 9. **MoCA** = Montreal Cognitive assessment. 10. **CDRS** = clinical dementia rating scale. 11. **HKLLT** = Hong Kong list learning test. 12. **CGDS** = geriatric depression scale. 13. **BAI** = Beck anxiety inventory. 14. **FP1** = left frontopolar region. 15. **FP2** = right frontopolar region. 16. **Pz** = peripherial zone. 17. **MCI = **mild cognitive impairment. 18. **HbO** = oxygenated hemoglobin. 19. **F7** = frontal region 7. 20. **AF7** = mastoid frontal region 7. 21. **Fpz** = prefrontal zero region. 22. **AFz** = mastoid frontal zero region. 23. **Fz** = frontal zero region. 24. **NPI** = neuropsyvhiatric inventory. 25. **CBF** = cerebral blood Flow. 26. **ATP** = adenosine triphosphate. 27. **DLPFC** = dorsolateral prefrontal cortex. 28. **MMSE** = mini-mental state examination. 29. **HAM-A** = Hamilton anxiety rating scale. 30. **HDRS** = Hamilton depression rating scale. 31. **CCO** = cytochrome C oxidase. 31. **HbR** = deoxygenated hemoglobin

## Results

### Study characteristics

The 37 articles selected showed different methodologies, including sample (human, animal, healthy aging, or disease), PBM parameters, and brain and behavioral assessment. We divided the studies into those that employed animal sample (Table 1), and those with human sample (Table 2).

### Animal studies

#### Sample characteristics

Twenty-three articles (62%) were performed with animals.

Of those, 30% employed healthy aged rats between with 16 [25, 26] and 20 months old [9, 27–30], 17% used healthy aged mice with 12 months [31], 18 months [32] or with an aging-induced protocol [33, 34], 39% employed an AD mice model [35–43], 9% a PD rat model [44, 45], and 4% used a postoperative neurocognitive disorder (PND) mice model with 18–20 months [46]. Considering sex, most of the studies (74%) were performed only in males [9, 25–31, 33–37, 39, 45, 46], 13% in females [38, 42, 44], 4% included both sexes [41], and 9% did not report the sex of animals [40, 43]. Most of the studies were methodologically cross-sectional, while 17% performed longitudinal analysis at ages 3, 7, and 12 months [38]; 3 until 9 months [37]; 4 until 6 months [39]; 1 until 6 months [41]. Regarding controls, all studies included at least a treatment control (the aged group or disease group with sham PBM), while some of them (30%) included a young control group, with 2 [25, 26, 32], 3 [29, 31] and 4 months [27, 31] of age.

### PBM parameters

In this section, parameters such as wavelength (nm), wavelength type (continuous or pulsed), frequency, intensity, irradiance, brain target area, days of application and usage of anesthesia were analyzed.

Regarding the PBM wavelength, 70% of the studies used NIR light, at 808 nm [37, 43, 44], 810 nm [9, 27–29, 33, 41, 46], 830 nm [25, 26, 45], 1070 nm [42], 1072 nm [38], and at 1267 nm [39]. Other studies (26%) employed wavelengths in the red spectrum with 610 nm [36], 630 nm [35], 635 nm [40], 660 nm [30, 32], and 670 nm [31]. Only one (4%) compared both an NIR light at 810 nm and a red light at 660 nm [34]. Considering wavelength type, 44% used a continuous wave [9, 25–30, 32, 45, 46], 17% pulsed at 10 Hz [33, 34], 100 Hz [41], and 600 Hz [38], while others 9% compared both continuous wave with pulsed at 40 or 100 Hz [37, 43], and 4% between 10 and 40 Hz [42]. Notably, 26% of the studies did not report whether the wave was continuous or pulsed [31, 35, 36, 39, 40, 44].

The 13% used a total intensity of 71.96 J [25, 26, 45], 22% 15 J -3 J per point [9, 27–30], 4% used 16 J/cm^2^ [32], 4% used 2.0 J/cm^2^ [36], 4% 4.5 J/cm^2^ [42], 4% 6 J/cm^2^ [40], 17% compared different intensities, such as 4 and 8 J/ cm^2^ [34], 16 and 32 J/ cm^2^ [39], 16 and 36 J/cm^2^ [33], and 80 and 160 J/ cm^2^ [46]. The 30% did not include data about intensity [31, 35, 37, 38, 41, 43, 44]. Regarding irradiance, 35% reported a value of 100 mW [25–30, 45], 4% reported 5 mW [38], 9% 200 mW [32, 34], 4% 8.75 mW [40], 4% 1 W [35], 4% 80 mW/cm^2^ [46], 4% 50 mW/cm^2^ [43], 4% 25 mW/cm^2^ [42], 4% 4.75 mW/cm^2^ [33] and 4% 1.7 mW/cm^2^ [36], 13% compared different irradiances, one with 40, 200 and 400 mW [37], another with 5 and 470 mW [41] and the last one with 2.5, 5 10, 25 and 30 mW/cm^2^ [44]. Finally, 9% did not report the irradiance employed [31, 39].

As for the selection of the brain area to apply the PBM, 17% used six irradiation points across the brain [25, 26, 38, 45], 22% used five irradiation points (Table 1 shows coordinates) [9, 27–30], 4% two irradiation points [36], 22% one irradiation point [32, 33, 37, 39, 44], 17% explained PBM was applied above the head [34, 40, 41, 46], 13% employed a transcranial application in a box [35, 42, 43], and 4% did not report brain target area [31]. The 65% of the studies applied PBM consecutively once a day for 3 [25, 26], 5 [35, 46], 7 [43], 10 [30], 14 [32, 45], 30 [40], 58 [9, 27–29], 60 days [42], and 8 consecutive months [31]. However, the 35% of studies applied PBM for non-consecutive periods, with different procedures: 7 days, once every two days [39], 18 days 3 times per week [34], 22 days in a period of 14 consecutive days, 6 weeks withdrawal, and 8 consecutive days [44], 24 days 3 times per week [33], 40 days 2 times per week [38, 41], 48 days 3 times per week [36], 72 days 3 times per week [37]. The longest application was 8 months [31], and the shortest 3 days [25, 26]. Finally, there is diverse anesthesia practices among researchers, as 17% of the studies utilized anesthesia during the treatment sessions [36, 37, 39], while the 78%, opted for non-anesthesia protocols [9, 25–30, 32–35, 38, 40–46]. Notably, 4% of the studies did not report their anesthesia usage [31].

### PBM effects on behavior

Regarding effects at a behavioral level, sixteen out of twenty-three (70%) of the animal studies aimed to study the behavior, including cognition, emotion, or locomotor activity. We have independently considered studies performed on healthy aging and on disease. First, we focus on cognition, then on emotional state, and finally, on locomotor activity.

As for studies conducted on healthy aged subjects, the 44% (four out of nine) focus on memory. Results reflected improvements in spatial learning and memory using both NIR [9, 33, 34], and red wavelenghts [32, 34]. Most of them employed the Barnes Maze [9, 32, 34], while one study used the Lashley maze [33]. Also, one study included an episodic-like paradigm, using the What-Where-Which task, and observed an improved performance in aging induced mice [32]. However, no differences were found in aversive associative memory using an inhibitory avoidance procedure [9], or a passive avoidance test [33]. Regarding social memory, although PBM did not alter a preference for social interaction, it has been observed an increase in the social memory index of aged mice following PBM [33]. It is important to note that these studies differ in days of application (from 10 to 28 total days), wavelength type (continuous or pulsed), intensity and irradiance. Notably, the studies which compared the behavioral results regarding intensities observed improvements with 8 J/cm^2^ [33, 34], and 16 J/cm^2^ [33], but not with 4 J/cm^2^ [34] or 32 J/cm^2^ [33].

Regarding AD models, the 89% (eight out of nine) examined memory performance. Results showed improvements in spatial learning and memory with red [36, 40], or NIR wavelengths [37, 39, 42, 43], other did not found differences following PBM with NIR light [41]. Most of them employed the Morris Water Maze [36, 37, 39–43], and some studies the Y maze [40, 41]. Regarding aversive associative memory, using the passive avoidance test, no differences were found with red light [36]. As for recognition memory, it was found mixed results: some studies reported improvements in the novel object recognition test with NIR [39, 42], while others did not observe significant results with red [35] or NIR light [41]. Notably, the studies which compared behavior regarding PBM parameters observed positive results with continuous or pulsed waves (40, 200, 400 mW), where 200 mW pulsed group seem to achieve the better performance [37]. Also, it has been shown comparable results with 808 nm and continuous or 40 Hz wave, as well as with visible light [43]. However, others found positive results only with higher intensity (32 J/cm^2^), when compared with 16 J/cm^2^ [39], or with 10 Hz in comparison with 40 Hz [42]. Additionally, cognition has been explored in PND and in a sleep-deprived (SD) model. The PND study showed that the treatment ameliorated the cognitive dysfunction in spatial learning and memory using the Barnes Maze, and in memory recognition. This study compared between 80 and 160 J/cm^2^, with no major differences [46], and was the unique study concerning this pathology. On a SD model, 50% of the studies (a total of two) aimed to evaluate cognition and observed that PBM did not ameliorate its effect on short term spatial memory on the old group [25].

PBM has been explored in anxiety, both in health and disease, although there is a scarcity of studies, with mixed results. One study on healthy aging (11%, one out of nine), assessed in the elevated plus maze, revealed no differences [9]. Interestingly, in the AD model only one study (11%, one out of nine) assessed this aspect and observed a reduced anxiety phenotype compared to a wild type was found, which was reversed by early PBM [36]. On the SD model 50% of the studies (one out of two) attempted this question and showed the PBM improved anxiety symptoms on aged rats [25].

Finally, according to locomotor activity studied in healthy aging, 22% of the studies (two out of nine), observed no alterations in the open field test [9, 32]. Similar results have been found in the AD model [35], with only one study out of nine (11%) including the measure. In PD, 100% of the studies (a total of two) evaluated motor activity, and observed PBM restored locomotor activity, both in the open field [45], or in the cylinder test to evaluate akinesia [44]. The first study observed improvements in the number of squares crossed, time spent in the central area, and freezing time, while not in rearing [45], and the second one reflected the treatment with 2.5, 5 and 10 mW/cm^2^ improved motor deficits, but not with 25 or 30 mW/cm^2^ [44].

### PBM effects on brain activity

PBM effects on brain activity were performed in all the studies included, with different levels of approach. We have independently considered studies performed on healthy aging and on disease.

In healthy aging, NIR light resulted in a reversion of the high concentration of several metabolic pathways to the levels of youngsters in the cerebral cortex [27]. Specifically, a decrease was observed in aspartate, glutamate, ammonia recycling, urea cycle, purine metabolism, arginine, proline metabolism, alanine metabolism, phenylalanine, tyrosine metabolism, glutathione metabolism, phosphatidylcholine biosynthesis, and glycine, serine metabolism [27]. Also, in the hippocampus PBM increased the concentration of acetate and guanosine triphosphate [27]. Furthermore, it has been studied the effect of PBM on brain metabolic activity through CCO histochemistry, revealing that the treatment with NIR light can reverse the decrease of metabolic activity associated with age [29]. Changes have been found in the ventral basal thalamic nucleus-lateral, the caudal caudate-putamen, and the nucleus of cranial nerve 3, CA1 of the hippocampus, mammillothalamic tract, auditory cortex, and primary and secondary visual cortex, whereas a reduction in anterodorsal thalamic nucleus, which shows a higher brain metabolic activity associated with age [29]. Moreover, red and NIR PBM with 8 J/cm^2^ enhanced the active mitochondria, mitochondrial membrane potential, CCO activity [34], and ATP levels with NIR PBM and 8 J/cm^2^ [34], and red PBM with 16 J/cm^2^ [32], while no differences were found with NIR PBM and 8 J/cm^2^ [34]. Similar results have been observed in a SD model in aging, showing increased CCO activity following NIR PBM treatment [26].

Effects on signaling proteins in the cerebral cortex revealed NIR PBM application led to increases in signal transducer and activator of transcription 3 (STAT3), extracellular-signal-regulated kinase (ERK), and c-Jun N-terminal kinases (JNK) in the cerebral cortex. However, no differences were observed in protein kinase B (Akt), ribosomal protein S6 kinase beta-1 (p70S6K), STAT5, and p38 [28]. In the hippocampus, increases in the expression of p70S6K, STAT3, and Akt were reported, with no differences in STAT5, ERK, JNK, and p38 [28]. Notably, red light reduced the cortical expression of ERK and p38 while increased the activation of STAT3 and ERK in the hippocampus [30].

Regarding oxidative stress, both red and NIR PBM at 8 and 16 J/cm^2^ led to reductions in reactive oxygen species (ROS) levels [34]. Additionally, in the SD model, it has been observed a reversion of superoxide dismutase both in the hippocampus [25] and in the hypothalamus [26], a hippocampal reversion of malondialdehyde (MDA) levels [25], and a hypothalamic reversion of glutathione (GSH) [26]. As for apoptotic proteins, including the Bax to Bcl-2 ratio and caspase 3, they were attenuated following treatment [34]. Effects on anti-apoptotic markers and neurotrophic factors revealed PBM with NIR wavelengths up-regulated anti-apoptotic markers such as Bcl-2 and increased the expression of brain-derived neurotrophic factor (BDNF) in the hippocampus of SD-aged rats [25]. Increases in BDNF and Bax were found in the hypothalamus, along with a reversal of hippocampal cholinergic neurotransmission (acetylcholine and acetylcholinesterase) [25].

Concerning neuroinflammation, PBM with decreased cortical levels of pro-inflammatory cytokines, such as IL-5, with both NIR [9] and red wavelengths [30]. Also, it has been found that NIR PBM can increase cortical IL-6, IL-10, and tumor necrosis factor alpha (TNFα) [9] and red wavelengths generates increments in IL-1α [30], while no differences were found in granulocyte–macrophage colony-stimulating factor, monocyte chemoattractant protein, and lipopolysaccharide-induced chemokine in cortical regions [9]. As for hippocampal measures, it has been observed a reduction of interferon gamma-induced protein (IP-10), fractalkine levels [9], TNF-α and IL-6 [33] with NIR, as well as reductions of IL-5, IL-18 and fractalkine levels with red PBM [30]. Notably, it has been shown that NIR PBM in the SD model is able to reduce TNF-α, IL-6 and c-reactive-protein [26]. Moreover, red PBM light lead to changes in glial cell number and morphology. It has been shown that PBM restores astrocyte reactivity associated with aging, reaching the levels of younger rats, and reduces microglia activation in the striatum [31]. Morphological analysis revealed that the PBM treatment in aged rats led to an astrocyte morphology like youngsters, with differences with the non-treated aged group, which showed larger and more strongly labeled astrocytes, indicating activation [31]. As for microglia, a 50% of reduction of activated microglia was observed following treatment, with no differences in morphology, suggesting a microglia resting-state morphology [31]. In neurons, no major changes were found after PBM application with red light either in parvalbumin or in encephalopsin interneurons in the striatum, nor in striatal dopaminergic terminals [31]. In SD rats, no differences were found in the maladaptive histoarchitecture of hippocampus treated with PBM [25], but there was a mitigation of the SD-induced alterations and restored the normal histological features of hypothalamus tissue [26]. Interestingly, other studies included synaptic markers and revealed that NIR PBM inhibited the downregulation of growth-associated protein (GAP-43) and synaptophysin (SYP) with 8 and 16 J/cm^2^, while no differences were found regarding post-synaptic density-95 (PSD-95), or with 21 J/cm^2^ of intensity [33].

In AD models, 89% of the studies (eight out of nine) studied amyloid proteins with red or NIR PBM [36–43], and except [41], all the studies found a decrease in amyloid proteins. There was a generalized reduction of Aβ plaques [37, 43], a reduction Aβ in the cortex [36, 39, 40, 42], and the hippocampus [38–40, 42], where it has been also observed a reduction of the amyloid precursor protein [38]. Also, there were increased levels of sAβPPα proteins [37]. However [36] did not found positive effects in the hippocampus, and, notably, the Aβ plaque reduction was observed when PBM application started at 2 months of age (early intervention) but not at 6 months (delayed intervention). To note, one study observed increments in the insulin-degrading enzyme in the cortex [36], and reduced p-JNK and c-Jun signals around the plaques [40]. Interestingly, a study which compared between continuous and pulsed PBM revealed only pulsed wave at 400 mW and 200 mW decrease amyloid load in the cerebrospinal fluid [37]. Comparisons between 10 and 40 Hz NIR light revealed 10 Hz reduced Aβ load in CA1 with 12 months, and both frequencies in the cortex with 6 and 12 months. Number of plaques were reduced with 40 Hz in CA1, and with both frequencies in the cortex of mice with 12 months [42]. Furthermore, PBM mitigated Aβ burden in the brain by improving lymphatic clearance of Aβ and increased diameter of the basal meningeal lymphatic vessels [39].

As for intracellular signaling, red PBM resulted in an enhancement of the mitogen-activated protein kinase phosphatase 7 phosphorylation, while inhibited JNK3 and PSD-95 phosphorylation and the AMPA receptor endocytosis [40]. Also, it has been observed an upregulation in members of the heat-shock protein signaling pathways (HSP60, HSP70, HSP105, HSP27-P, HSP27, PS1) [38].

Additionally in AD models, PBM with NIR leads to a reduction in inflammatory markers such as IL-1β, TNF-α, and TGF-β, regardless continuous or pulsed waves with 40, 200 and 400 mW [37]. Also, a reduction in degenerating neurons was observed at both early and delayed treatments [36], and rescues in the decrease of dendritic spines [40]. Others did not observed differences in neuronal loss [41]. It was revealed that early PBM reduced microgliosis in the cortex in the AD model [36], but no differences were found in the hippocampus [36], or with not earlier treatments [36, 41]. Furthermore, 10 Hz increased the colocalization between microglia and Aβ in the cortex of the mice, while there was no difference in the astrocytes, and it reduced M1-like microglia [42].

Positive effects have been found in synaptic function and plasticity of AD studies: PBM with red light resulted in rescued field excitatory postsynaptic potential, long-term potentiation, and partially restored long-term depression, but did not modify paired-pulse facilitation [35]. Also, PBM with red light resulted in increased SYP and the microtubule-associated protein-2 (MAP2) in the cortex and hippocampus [40]. Finally in AD models, one study showed that PBM with NIR restored ATP levels in AD model as well as induced an increase in c-fos protein expression [37].

In PD models, NIR PBM decreased MDA, GSH, and nitric oxide (NO) in midbrain and striatum. It also recovered AchE, and monoaminoxidase enzymatic activity in midbrain, while no effects were found in Na + , K + -ATPase. Monoamines such as norepinephrine, and serotonin were restored, while no significant differences were found in dopamine, in the midbrain and striatum [45]. Furthermore, NIR PBM with different conditions (5, 10, 20, 25, 30 mW/cm^2^) showed less nigral dopaminergic degeneration with a significant protection against α-syn-induced toxicity on the highest fluence group as well as less striatal fiber denervation with a significant effect observed after treatment at higher fluence [44]. No differences were found in cell survival or cortical cell density [44]. Finally, applications of NIR PBM on PND decreased ROS and TNF-α. This study also found that PBM upregulated the interferon regulatory factor 7, reduced microglia M1 and increase M2 phenotype, upregulated the expression of BDNF, CCO and improved ATP production restoring enzyme activity of complexes I, II and IV. Furthermore, it altered the profiles of mRNA in the prefrontal cortex and hippocampus and reversed expression of inflammasome proteins [46].

### Human studies

#### Sample characteristics

Fourteen out of the thirty-seven articles (38%) were performed with human patients.

Of those, 43% were performed with healthy patients with ages ranging between 50 and 85 years [16, 47–51], 21% used patients with AD, and/or dementia [52–54] 14% involved MCI [55, 56], 7% examined both AD and MCI [57], and the last 14% examined PD [58, 59]. Considering sex, 93% of the studies were performed in men and women [16, 47–54, 57–59], whereas 7% used only women subjects [56], resulting in an average percentage of male participants of 34%. All the studies were cross-sectional, with no longitudinal explorations. Regarding controls, 64% of studies included a treatment control [16, 49, 51–57], 14% compared between two PBM protocols [58, 59] and 21% used pre PBM treatment as baseline control [47, 48, 50].

### PBM parameters

In this section, the same parameters as in Sect. 3.2.2 are included, except for the use of anesthesia.

Regarding the transcranial PBM wavelength, 71% of the studies used infra-red light at 810 nm [48, 53, 55, 57, 59], 850 nm [56], 904 nm [58], and 1064 nm [16, 50, 51]. The 21% used red light, at 633 nm [52], 650 nm [54], and 670 nm [47], while 8% combined both 633 nm 870 nm [49]. Considering wavelength type, 36% of the articles used a continuous wave [16, 48, 49, 51, 55], while 21% employed pulsed at 40 Hz [53, 57, 59], 7% at 50 Hz [58], while 36% did not report whether the wave was continuous or pulsed [47, 50, 52, 54, 56].

With respect to intensity, 7% of the studies utilized 3.4 J [50], 7% 42 J [58], 7% 60 J [56], 7% 120 J [16], 7% 240 J [59], 7% 300 J [57], and 28% did not report the intensity [47, 49, 52, 54]. As for the irradiance 7% used 4 mW [52], 14% 20 mW/cm^2^ [48, 55], 7% 60 mW/diode [58], 7% 100 mW [53], 7% 150 mW/cm^2^ [57], 14% 200 mW/cm^2^ [59], 21% 250 mW/cm^2^ [16, 50, 51], 7% 400 mW [56], 14% 999 mW [49], while 14% did not report the intensity [47, 54].

Other aspect to consider is the application method, were 79% of the studies employed transcranial application with 1 [16, 47, 50, 51, 56], 2 [53], 3 [49, 57], 4 [58], and 9 irradiation points [48, 55], which varied from studies (see Table 2). Also, 7% of the studies utilized intranasal application [54], 7% utilized intravenous application [52] and 7% combined transcranial, intranasal, transdermal and transabdominal [59]. Finally, as for treatment duration, 36% of the studies applied PBM for one day [48, 49, 51, 55, 56], 7% for 4 consecutive days [47], and 14% for 7 consecutive days [16, 50]. For a longer treatment duration, 14% of the studies applied PBM for 36 non-consecutive days, 3 times per week [53, 54], while another 7% implemented a treatment duration of 72 non-consecutive days, with sessions held 6 times per week [57]. Furthermore, 7% of the studies utilized a treatment regimen in 6 courses, implemented over an 18-month period [52], and 14% used mixed longer protocols: [58] had protocol 1 with a month of placebo treatment (3 times per week), followed by a month of washout, and then a month of PBM or placebo (3 and 1 times per week, respectively), and protocol 2 with month of PBM treatment (3 times per week), followed by a washout month, and then a month of PBM or placebo (1 and 2 times per week, respectively) [58]. Also, [59] applied PBM in protocol 1 during 144 non-consecutive days, starting with three sessions per week for 4 weeks, followed by two sessions per week for another 4 weeks, then one session per week for 4 more weeks, and finally three sessions per week for 40 weeks. In protocol 2, 99 non-consecutive days, following a similar pattern but with a shorter duration, including three sessions per week for the first 4 weeks, followed by two sessions per week for another 4 weeks, then one session per week for 4 more weeks, and finally three sessions per week for 24 weeks [59]. The longest application was 144 active days [59] and the shortest 1 day [48, 49, 51, 55, 56].

### PBM effects on cognition and emotional state

Twelve out of fourteen of the human studies (86%) included neuropsychological assessment. As in preclinical studies, we have independently considered studies performed on healthy aging and on disease. First, we focus on cognitive assessment, and then on the emotional state.

In healthy aging, 67% of the studies (four out of six) included neuropsychological assessments. It has been shown that PBM can improve working memory, assessed in the N-back task [16, 50], with NIR wavelength. Interestingly, results were maintained up to three weeks after PBM application [16, 50]. However, no differences were found after treatment in visual memory with NIR light [48]. Furthermore, healthy aged subjects improved cognitive inhibition and lexical/semantic access, evaluated with the modified Eriksen flanker test and the category fluency test, respectively, after a single application of combined red and NIR PBM. Faster reaction time was found during both the congruent and incongruent post-intervention conditions in subjects with PBM but not in the controls. A higher number of total words generated during the category fluency test was observed in treated subjects [49].

In AD or dementia, 100% (a total of four) of the selected studies included neuropsychological assessments, and observed improvements in cognition and neuropsychiatric symptomatology, assessed with Alzheimer’s Disease Assessment Scale-cognitive (ADAS-cog), the Neuropsychiatric Inventory (NPI), or the Montreal Cognitive Assessment (MoCa) [52–54, 57], with red [52, 54] and NIR wavelengths [53, 57]. Notably, the study of [52] compared different therapies (including light chromotherapy, magnetic field therapy, and pharmacotherapy), and observed similar results with all therapies, except from light chromotherapy. Also, one study included both AD and MCI patients [57]. Regarding effects of PBM on MCI patients, 100% of the studies (a total of two) assessed cognitive function. It has been found improvements in visual memory in the short-term [55], in cognitive status through mini-mental state examination (MMSE), and in attention (reduction in reaction time, increased correct trials, and efficiency score) [56]. Both studies employed NIR wavelengths. In PD, 100% of the studies also included the neuropsychological assessment (a total of two), with contradictory results. One study found significant improvements in the cognitive function evaluated with MoCa [59], whereas another did not observe positive results using the same assessment tool [58], both with NIR wavelengths.

Like what happened in preclinical studies, few articles included the assessment of the emotional state following PBM. In healthy aging, no improvements were found in anxious or depressive symptomatology in women [49]. In AD and MCI, it has been observed an improvement in the depressive symptomatology, but not in anxiety symptoms [57].

### PBM effects on brain activity

PBM effects on brain activity were examined in six out of the fourteen human studies (43%). As previously, we have independently examined studies conducted on both healthy aging and disease, distinguishing between conditions of basal activity and cognitive load.

In healthy aging and under basal conditions, it has been observed that NIR PBM application increased oxidized CCO in the right and left prefrontal cortex, and decreased deoxygenated hemoglobin (HbR) in the right prefrontal cortex [51], with no changes in prefrontal oxygenated hemoglobin (HbO). However, NIR PBM applied pre- and post-working memory assessment modified cortical hemodynamic activity leading to a decrease in HbO activation in the right hemisphere during the task, with an expansion from the first to the last day of the treatment, with changes remaining at the two-week post-stimulation [50]. Similar results were observed in a visual memory task, where decreases in HbO were associated with the difficulty levels of the task, but not with the easiest [48]. Regarding HbR, after one week of stimulation with PBM, the temporal response seemed to change, and hemoglobin concentration during the 3-back task increased in the right bilateral premotor cortex and right visual cortex, remaining at the two-week post-stimulation [50]. Interestingly [51] compared brain activity changes in older versus younger groups, observing a marked treatment-induced effect on CCO with increasing age, but a decrease in HbO [51]. Finally in healthy aging, one study revealed PBM increased ATP synthase flux under basal conditions [47].

Concerning dementia, it has been found that NIR PBM treatment under basal conditions increased the cerebral perfusion and connectivity between the posterior cingulate cortex and the lateral parietal nodes, and the cerebral blood flow (CBF) in the parietal cortex, but there was no significant difference in the default-mode-network activity between the groups [53]. Finally, regarding MCI, the NIR PBM therapy reduced frontal lobe HbO in response to the performance of a visual memory task, both in easier and difficult levels of the task [55].

## Discussion

In this review, our objective was to compile preclinical and clinical evidence from the last 20 years concerning the effects of PBM on aged individuals. We assessed the effectiveness of the therapy through an analysis of application parameters, behavioral and neuropsychological results, as well as brain-related modifications. The review included 37 articles.

### Sample characteristics in PBM studies during aging

It is noteworthy that nearly two-thirds of the investigations were preclinical studies, with the remaining carried out in human subjects. This distribution suggests that the application of PBM in aging-related conditions is still in its early stages, and further research is necessary to advance towards safe and effective treatments.

Regarding health-disease conditions, 47% and 43% (preclinical and clinical, respectively) recruited a healthy aged sample, 39% and 21% studied AD, while the rest were performed in other conditions (MCI, PD, and PND). In aging intervention, the primary focus could be on preventing and treating cognitive decline, with PBM emerging as a promising preventive tool that could be used in prodromal stages of neurodegeneration. In this line, one study applied PBM for 5 months from the prodromal phase of an AD model but did not observe a positive effect at a behavioral and brain level, suggesting potential methodological limitations (regarding parameters, or the sensitivity of the behavioral test, that could need to discriminate small cognitive changes)[41], underscoring the need to deepen into PBM mechanism of action. Additionally, the inclusion of AD models and human sample may be particularly relevant considering that this disease shares some mechanistic parallels with aging, notably in oxidative stress, and mitochondrial dysfunction [60, 61].

Considering sex, preclinical and clinical studies show disparities. While 93% of human studies included both men and women [16, 49–52, 54, 55, 58, 59, 62], most of the preclinical studies were performed exclusively in males (74%), with only a minority (17%) incorporating females into their investigations. Furthermore, some studies (8%) omitted specifying the sex of the animals used. This disproportionate representation underscores a prevailing issue within preclinical neuroscience research, where females are often underrepresented or overlooked (26% versus 5%, respectively) [63]. Addressing this issue is crucial for ensuring the validity and generalizability of research findings, as well as translation to clinical studies. Furthermore, the preclinical study which consider both sexes, do not present results considering sex an experimental variable -although groups were sex-balanced- [41] while only 7% of human studies did [54]. This becomes significant due to medical, genetic, hormonal, behavioral and psychosocial factors can differ in a sex-specific manner during aging [64–66]. Thus, it is necessary not only to include females or women, but also to present results considering this factor [63].

Finally, methodological designs and additional control groups must be considered. Most of the studies employed cross-sectional designs, with a lack of longitudinal studies, could provide crucial insights into the long-term effects of PBM and its interaction with disease over time.

### Parameters in PBM studies during aging: current status towards standardization

The selection of PBM parameters is related to light penetration and exert a different modulation on brain physiology and behavioral outcomes [67]. The compiled studies (preclinical and clinical, independently) reflect an elevated level of heterogeneity, and some studies lack for information, being difficult to establish solid conclusions.

Wavelength, as one of the key parameters influencing PBM outcomes, exhibits notable variability among studies. It is known that between 600 and 1200 nm CCO exerts an adequate absorption capability. It is because this enzyme presents red (620–689 nm) and NIR (760–825 nm) spectral absorption peaks [27], and the use of longer wavelengths, such as 1064–1072 nm, promotes photo-oxidation and triggers increased brain oxygenation [68]. The precise impact of different wavelengths is unclear, and it is suggested that PBM interaction with CCO is wavelength dependent. Following the absorption of photons by CCO and subsequent photo-oxidation, it occurs a cascade of cellular and physiological processes, including upregulation of CCO, leading to increased oxygen consumption, ATP production, NO release, and enhancement of mitochondrial membrane potential [67]. In this systematic review, we observed that most of the articles, both on rats and humans, used NIR light between ranges of 808 nm and 1267 nm [9, 16, 27–29, 37–39, 42–45, 48, 50, 51, 53, 55–59], being the most used 810 nm [9, 27–29, 33, 41, 46, 48, 53, 55, 57, 59]. The rest of the studies used red light between 610 and 670 nm [30–32, 35, 36, 40, 47, 52, 54], and some of them used a combination of both red light and NIR light [34, 49]. The highest selection of NIR light may respond to previous studies which outline 810 nm may be the optimal wavelength to be used [69]. Hence, some studies reflect that 810 nm wavelength exhibits the highest energy deposition, followed by the 850 nm and 1064 nm wavelengths, which deliver more energy than the 670 nm and 980 nm wavelengths [70]. However, it is important to note that simulation dosimetry studies revealed a decrease of energy deposition with increasing age [70], which reflect the need for age-specific adjustments in PBM dosages to ensure therapeutic efficacy [70]. Interestingly, [16, 38, 39, 42, 50, 51] employed longer wavelengths (from 1064 to 1267 nm), and a recent review signaled this selection (particularly 1064 nm) may be associated to reduced photon scattering, and although not being optimal in terms of mitochondrial absorption, it may exert greater penetration and targets light-sensitive ion channels [71].

Regarding type of wavelength, 36–44% of studies used continuous waves [9, 16, 25–30, 32, 45, 46, 48, 49, 51, 55], while 27–30% reported using a pulsed wave [33, 34, 38, 41, 53, 57–59], a 5% used both continuous and pulsed [37, 43], and 26–36% did not specify the wavelength type [31, 35, 36, 39, 40, 44, 47, 50, 52, 54, 56]. The predominant methodology can be against literature hypothesis which suggest pulsed wave -intermittent delivery of light-, may dissociate more NO compared to continuous wave -constant delivery of light- during photodissociation [72], which may have an impact in increasing the rate of respiration and ATP production [21]. Interestingly, comparative studies suggest enhanced benefits with pulsed wave in AD pathology [37], and cognitive enhancements with pulsed waves (40 and 100 Hz), when compared with continuous in young adults [73]. Also, regarding pulsed waves, selection of frequency is important. The singular effect of frequency can affect brain activity, modifying the frequency bands, such as alpha and theta waves [74]. Aging is associated with modifications in brain oscillations, and significant changes in resting-state electrical activity with disrupted brain connectivity appear in AD [75]. Thus, understanding the interplay between frequency and brain activity is essential. It is necessary to determine the timeframe in which PBM is biologically active, which is influenced by both PBM parameters and the physiological state of the subject.

It is proposed that PBM relies on a biphasic response, reflecting that it can induce either inhibitory or stimulatory effects by employing different energy densities. Low to moderate doses of light can stimulate beneficial cellular responses, such as the expression of protective factors. However, excessively high doses may lead to diminishing returns or even harmful effects [21]. When selecting energy deliver, it is essential to consider the age, or, if possible (and more desirable), the thickness of extracerebral tissues, which are the main factor affecting energy deposition. In this systematic review, it is observed a highly variability of this value, with some studies which do not include the data [31, 35, 37, 38, 41, 43, 44, 47, 52, 54]. Various articles reflect the total energy deliver (J), others the fluence (J/cm^2^), and others the irradiance (W). It is important to include all parameters to facilitate study replication.

**Fig. 2:**
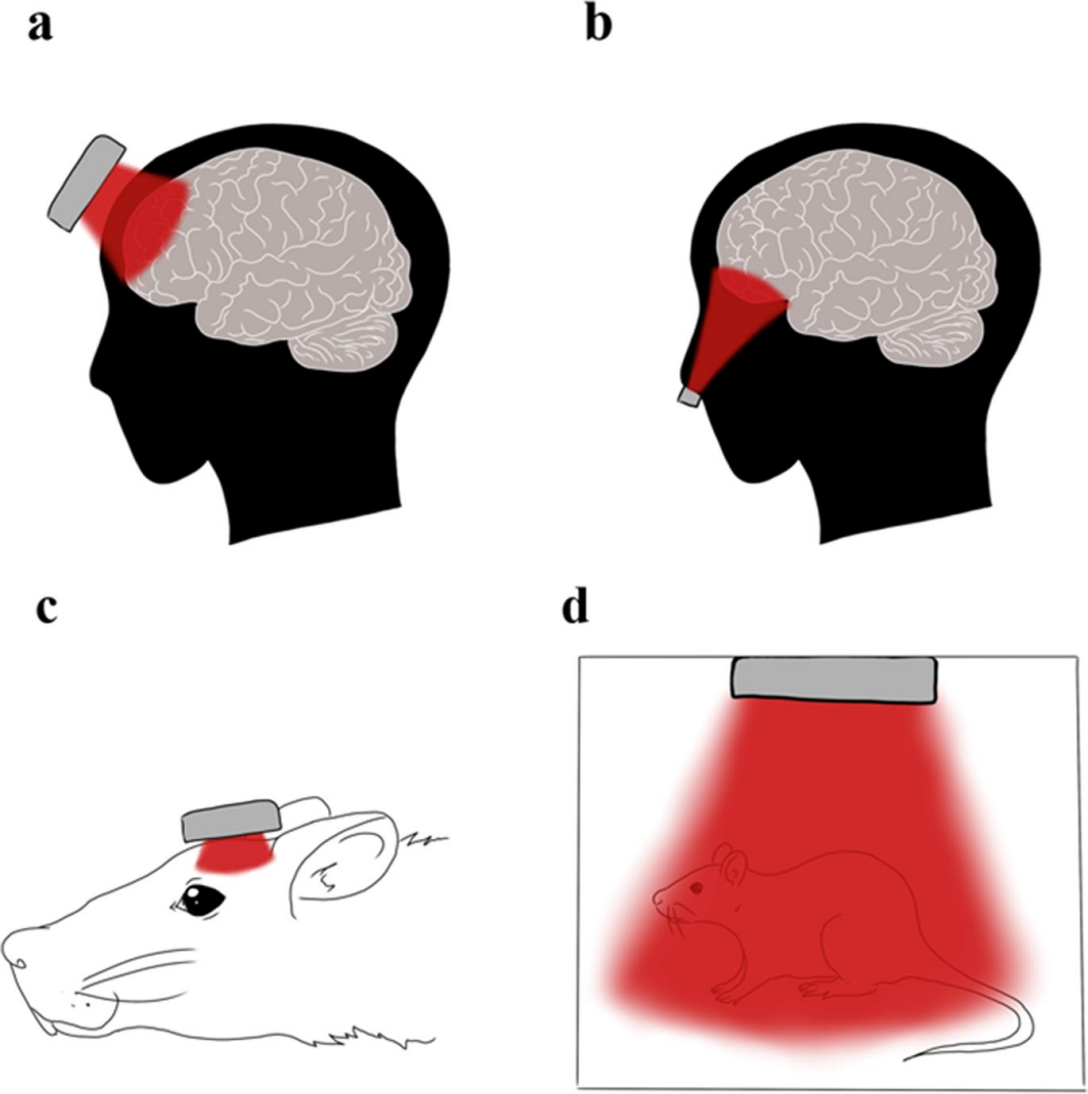
Schematic representation of PBM application. Image shows PBM transcranial (**A**) and intranasal (**B**) application on human patients. (**C**) shows a graphic representation of transcranial application and generalized in chamber (**D**) application of PBM in a rodent model

Additionally, the target region where PBM is delivered is important, not only due to functional aspects, but also due to absorption, which may vary according to distance between the beam source and the target. Understanding these parameters, alongside considerations of physiological and anatomical disparities, facilitates the selection of optimal parameters to maximize light penetration efficacy [67]. Among preclinical studies, diverse target brain regions have been observed, with applications addressed across most of the cortical surface using anesthesia [36, 37, 39], while others perform a more generalized application, either over the head or using a chamber [9, 25–30, 32–35, 38, 40–46]. Regarding humans, many studies applied it over the frontal cortex [16, 48–51, 54–56, 59], while others included the occipital, parietal, and temporal cortices [47, 53, 57, 58]. Additionally, some protocols combine transcranial application with intranasal, transdermal, intravenous or transabdominal approaches [52, 53, 59], suggesting the synergistic effect of PBM. Figure 2 shows a representative scheme of the main application PBM modes. Key characteristics of brain aging include loss of gray and white matter volume, cortical thinning, widening of sulci, and ventricular enlargement. Notably, the rates of gray matter atrophy vary between sexes, with men showing an annual loss of 0.424% and women of 0.298%, highlighting the relevance of study inter-individual differences, and consider sex. Additionally, gray matter atrophy has been proposed as a biomarker to differentiate between healthy aging and AD progression, where hippocampal and entorhinal cortex atrophy are pivotal indicators [76]. In this line, PBM shows promising results reaching hippocampal regions, as [9, 27, 28, 32, 39, 40, 42, 46, 77] report. Finally, time and days of application are highly variable across studies, ranging from one day [25, 26, 35, 39, 46–49, 51, 55, 56] to several weeks or months of treatment [9, 27–34, 36–45, 52–54, 57–59], which underscores the need for standardized protocols to assess the temporal effectiveness of PBM, which naturally may correlate with the varying amounts of energy applied. Also, both preclinical and clinical studies differ from consecutive or non-consecutive applications. Most rodent studies applied PBM consecutively once a day for periods ranging from 3 days to 8 months [9, 25–32, 35, 40, 42–46], while approximately 35% of the studies applied the treatment in non-consecutive periods, using various procedures such as alternate days, several times per week, or with interspersed withdrawal periods [33, 34, 36–39, 41]. In humans, approximately half of the studies applied consecutively from one to seven days [16, 47–51, 55, 56], while the rest used different non-consecutive protocols, from one to 18 months [52–54, 57, 58, 78]. This wide range of durations could impact the observed results and the interpretation of the effects of PBM on healthy aging and disease.

To sum up, the PBM efficacy is influenced by various parameters such as wavelength, energy delivery, and application method and duration. Despite this diversity, certain trends emerge. For instance, a significant proportion of studies utilized wavelengths around 810 nm for both rodent and human models. Additionally, while continuous-wave PBM was common, there was also a notable use of pulsed-wave therapy. Standardized protocols are necessary to assess the temporal effectiveness of PBM and optimize its therapeutic benefits.

### Behavioral and neuropsychological assessment in PBM studies during aging

Most preclinical studies (70%) included analysis of PBM effects on behavior, and 86% of clinical studies conducted neuropsychological assessments, with percentages varying from health to disease conditions.

Preclinical research shows improvements in spatial memory [9, 32–34, 36, 37, 39, 40, 42, 43, 46], episodic-like memory [34], recognition memory [42, 46], and social memory [33], while no effects were found in short- and long-term aversive memory [9, 33], both on healthy aging and disease. This may be due to the nature of the task, as aversive memory elicits a robust emotional response which may be more difficult to modulate by external interventions, in compassion with others, such as spatial memory [79]. To note, some studies on disease did not found improvements in spatial memory [41] and recognition memory [35, 41]. Therefore, the mixed findings in studies on disease highlight the complexity of memory-related disorders and the potential variability in individual responses to PBM treatment. Notably, these effects were observed across a range of PBM parameters, including different wavelengths, intensities, and durations of treatment. In humans, several studies suggest that PBM can improve various aspects of cognition, including working memory [16, 50], inhibition ability and language improvements [49], while no effects were found in visual memory [48]. Beneficial results have been observed not only in healthy older adults but also in individuals with AD and MCI [52–57]. The reported improvements in cognition in AD patients, as measured by the ADAS-cog scores and other neuropsychiatric assessments, are particularly noteworthy, as they suggest a potential therapeutic benefit of PBM in mitigating cognitive decline in this population, suggesting beneficial effects on cognitive function and quality of life. Additionally, while some studies report significant improvements in cognitive function in patients with PD [59], others find no significant changes or inconsistent results [58]. This discrepancy may be due to variations in disease severity, duration of treatment, and individual differences in response to PBM.

The observed improvements in memory function may be influenced by various factors, including study design, disease stage, severity, and comorbidities that may interact with PBM effects. Also, as noted before, the targeted brain region is important. Preclinical studies are less specific than clinical, and in human studies, while the prefrontal region, implicated in higher cognitive functions, has been the primary focus of investigation, fewer studies have explored PBM effects on other brain areas, such as the parietal cortex, which is linked to the spatial representation of episodic information and serves as a pivotal area in memory-based spatial navigation [80–82]. Additionally, the prefrontal cortex has been intricately associated with the hippocampus, demonstrating interactions in memory processes. This interplay is pivotal for memory consolidation [83]. It is worth noting that some animal studies did not specify a particular brain region as a target and instead irradiated the entire organism. Additionally, many of the studies rely on small sample sizes and lack long-term follow-up assessments, which limit the generalizability and reliability of the reported findings.

Anxiety manifestations among the elderly are frequently encountered and can significantly disrupt daily functioning, representing a clinically significant issue [84] particularly when occurring alongside other age-related conditions such as dementia. In the case of AD, anxiety often co-occurs with depression during the initial stages of the disease [85]. PBM has been used in clinical studies of anxiety and depression with positive results in both human and animal adults [17]. Given the rising prevalence of anxiety in older populations [86], there is a need to find efficient ways to treat it. Regarding the potential use of PBM in treating anxiety, the scarcity of animal literature on this topic underscores the need for more research. While one study suggests a reduction in anxiety in an AD model (5xFAD mice) and normalized anxious responses following PBM treatment [36] the limited evidence highlight the need of further investigation. The early endophenotypes of anxiety and depression in AD [87] outline the importance of exploring PBM as a potential intervention in the prodromal stages of the disease. Additionally, some studies observed a reduction in anxiety [25], while others did not found differences [9]. Differences between these studies may be due to the sample characteristics, where both studies included aged subjects, but [25] adds SD, a well-known stressor [88]. Hence, studies with accused alterations in anxiety (posttraumatic stress disorder) show PBM can prevent it with early interventions [89], which suggest PBM can be useful when severe symptomatology. In humans, few studies related these issues. Some studies have indicated that applying PBM to the right prefrontal cortex resulted in reductions in depressive symptoms among human subjects [90–92]. However, conflicting findings have been reported, with some studies failing to observe significant effects on depressive and anxiety symptomatology in individuals over the age of 60 [49]. In terms of anxiety treatment, a preliminary study revealed that adult patients with generalized anxiety disorder experienced a notable reduction in symptoms following PBM therapy [93].

Finally, investigations into locomotor activity have consistently shown no discernible alterations attributable to PBM treatment [9, 32, 35], adding information about the safety of PBM at a behavioral level. Notably, in PD models, PBM led to significant improvements in locomotor activity, with sustained benefits even after cessation of treatment [59]. Dysfunction in mobility has been related not only to cognition but also to the ability to integrate sensory information [94].

The positive findings of the improvement in cognitive functions after the PBM treatment in healthy adults, are a promising way to improve the normal cognitive decline that occurs in aging [16, 49, 50, 90–92]. The findings underscore the complexity and variability of PBM effects on cognition and emotion, with mixed results observed across preclinical and clinical studies. While some research indicates promising improvements in memory and cognitive function, inconsistencies in study design, outcome measures, and targeted brain regions highlight the need for standardized protocols and further investigation. Further studies are needed, considering that most of the research regarding cognition has been applied in adulthood. Finally, it seems that PBM is a promising early intervention in some diseases like AD, PD, or MCI, aimed at slowing down the deterioration of the cognitive process and improving people’s quality of life [52–56, 58, 59, 95, 96].

### PBM effects on brain activity during aging: a molecular, cellular and network level approach

In preclinical assessments, the studies here included reveal the beneficial effects of PBM across different experimental paradigms, shedding light on its potential therapeutic applications in healthy aging, and in aging-related neurological disorders. 100% of animal studies and almost half of the human studies (43%) [47, 48, 50, 51, 53, 55] examined the brain-related modifications.

In the context of healthy aging, in both animal and human studies, PBM demonstrates promising effects in reversing metabolic alterations, which may be associated with aging. There is evidence of restoration of CCO activity [26, 29, 34, 46, 51], indicating improved mitochondrial function in both contexts. Preclinical studies indicated that PBM can reverse metabolic alterations associated with aging in the cerebral cortex, restoring concentrations of key metabolites to levels comparable to younger counterparts [27], whose abnormal levels are linked to neuronal degeneration. For example, it has been found that a dysregulation of purine is associated with AD, inflammation, and neuropsychiatric disorders, among others [97, 98]. The restoration of the aged rats’ metabolic pathways and the increase of young rats’ metabolic pathways that are implicated in cortical excitatory neurotransmission and oxidative metabolism associated with CCO [99, 100] coincides with the findings of other studies of PBM-induced neuroprotection, anti-inflammatory, neuromodulatory, and antioxidant effects [101, 102]. PBM demonstrated a significant impact on CCO activity in various brain regions with metabolic alterations associated with aging, further supporting its potential as a modulator of cellular metabolism and energy production [26, 29, 34, 46]. Moreover, studies investigating ATP levels in the hippocampus following PBM applications have provided compelling insights [32, 34]. These studies align with the findings regarding CCO activity, suggesting that the modulation of PBM on CCO activity may contribute to the observed changes in ATP levels. It has been revealed enhancements in the active mitochondria, mitochondrial membrane potential and ATP levels [32, 34, 37, 46]. The restoration of ATP levels in the hippocampus following PBM treatment underscores the role of enhanced mitochondrial function, facilitated by increased CCO activity, in promoting cellular energy production and metabolic balance [21, 103–105].

In healthy older human adults, [51] observed an increase in oxidized CCO in the prefrontal cortex following a single session of PBM, particularly notable in the older age group. Various studies found reductions in HbO activation [48, 50], or in HbR [51], revealing changes in brain oxygenation levels, mainly accompanied by significantly improved task accuracy. Similar results have been previously observed in adults or adolescents [106, 107], suggesting enhanced neural efficiency leading to decreased cognitive load during complex tasks. Interestingly, the hemodynamic modulation changes over time, and it is possible to extend the reduction of HbO brain activation from one to both hemispheres [50]. It has been proposed that a repeated one-week stimulation likely increased energy deposition, leading to a greater number of photons interacting with cerebral tissue, thereby activating the brain from localized regions to brain networks [50]. This effect persisted even two weeks post-stimulation, which is particularly valuable in clinical settings. Connectivity network assessments demonstrated increased brain-wide functional connectivity and global small-world efficiency following PBM application, suggesting the potential to counteract age-related cognitive decline and reorganize cortical networks towards a more youthful state [108, 109]. Furthermore, investigations into conditions like AD and MCI reveal similar results. In individuals with AD and MCI, PBM therapy led to a reduction in the HbO during a visual memory task, suggesting a potential modulation of brain activity associated with cognitive decline [48, 55]. Also, in AD, [53] found increased cerebral perfusion and connectivity following PBM treatment, which are consistent with previous research performed in adults [110]. AD is characterized by decreased CBF and glucose metabolism, and enhancements of CBF have been previously reported in adulthood after traumatic brain injury [17], suggesting PBM as a promising intervention tool. These results hold clinical significance, potentially optimizing oxygen and nutrient delivery to brain tissue, thus benefiting neurological conditions like neurodegenerative diseases and brain injuries.

It is important to note that brain endothelial cells play a crucial role in regulating cerebral blood flow, the blood–brain barrier, and the response to brain inflammation, among other functions [111]. Notably, PBM may confer benefits to -microvascular endothelial cells by increasing cell proliferation and potentially protecting them against inflammation-induced apoptosis [112], suggesting a potential application in cases of endothelial dysfunction. Also, studies performed in humans show PBM can promote metabolic connectivity and affect hemodynamic-metabolic coherence in the prefrontal cortex, suggesting beneficial effects on brain-microvascular endothelial cells and neuronal function [113]. However, it is important to acknowledge that cerebrovascular dysfunction plays a role in the development of age-related cognitive decline. Some studies have shown deficits in neurovascular coupling [114, 115], while frontal networks exhibit stronger local and global connectivity. This may suggest that in the elderly, stronger functional connections are needed to compensate for the weakened neurovascular coupling during the performance of a higher demanding task [115].

PBM treatment was found to modulate intracellular signaling proteins without altering their expression levels, suggesting a fine-tuned regulatory effect on signaling cascades crucial for cellular homeostasis [28, 30]. Hence, PBM led to increases in ERK and JNK [28, 40], indicating critical roles in the regulation of glucose metabolism [116]. Also, STAT3 signaling -decreased during aging- was up-regulated, outlining cell survival, proliferation, and differentiation by modulating the expression of anti-apoptotic genes of the Bcl-2 family [28]. These findings align with similar results obtained with 660 nm PBM treatment, reflecting a consistent and reproducible modulation of brain responses across different wavelengths [30].

Furthermore, PBM exhibited immunomodulatory effects, reducing levels of inflammatory cytokines while enhancing the expression of anti-inflammatory markers in the cortex and hippocampus of aged rats. Specifically, chronic application of PBM resulted in a reduction of IL-5 levels in the cortex, indicating a suppression of pro-inflammatory signaling pathway [9, 30]. Others found a reduction in hippocampal or hypothalamic IL-6 and TNF-α [26, 33]. Concurrently, an increase in levels of other inflammatory markers, including IL-6, IL-10, and TNF-α, was observed [9]. It has been proposed that IL-6 exerts pro- and anti-inflammatory properties, and PBM could be restoring the reduction often found during aging. Similar, reduced levels of IL-10 during aging may respond to vulnerability and neuronal dysfunction, which could be reversed by PBM [9]. Also, the observed decrease in IP-10 and fractalkine levels in the hippocampus of PBM-treated rats further supports the anti-inflammatory effects of PBM [9]. Glial cell reactivity increases with aging, reflecting the intricate relationship between neuroinflammation and changes in both astrocyte and microglia morphology, and the aging process [117, 118]. Notably, PBM treatment resulted in a reduction in astrocyte and microglia reactivity, effectively restoring astrocyte morphology and microglia resting-state morphology to levels comparable to younger rats [31]. Overall, these findings suggest a potential role for PBM in attenuating neuroinflammation, a common feature of aging-related neurodegenerative diseases [119–121]. However, it is noteworthy that anti-inflammatory effects of PBM, as seen in animal studies [9, 26, 30, 33, 37], have yet to be fully validated in human research.

Other studies have focus on detrimental factors affecting aging, and its potential treatment with PBM, such as SD. Hence, PBM interventions have demonstrated effectiveness in enhancing antioxidant status, mitigating oxidative damage, suppressing neuroinflammation, and regulating CCO activity in the hypothalamic tissue of aged SD rats [25]. Also, PBM inhibited AChE, bolstered ACh production, and reduced ROS levels, indicating potential in enhancing cholinergic neurotransmission and alleviating oxidative stress associated with aging [26]. Additionally, PBM modulated apoptotic markers like Bax and Bcl-2, promoting cell survival and averting neuronal apoptosis in senile rats [26]. Finally, one study achieved attenuate neuroinflammation, enhance mitochondrial function, and promote neuronal survival in a postoperative neurocognitive disorder model [46], suggesting implications for the development of PBM-based interventions aimed at preventing or ameliorating PND in clinical settings.

Regarding animals with pathology, PBM treatment in AD shows promising results, unless the study of [41]. Several studies have investigated the effect of PBM therapy on reducing Aβ plaques, suggesting a consistent reduction in Aβ levels [35–40, 42, 43], and highlight the potential therapeutic role of PBM on neurodegeneration. The observed reductions in inflammatory markers suggest that PBM may exert its beneficial effects not only through direct Aβ clearance but also by modulating neuroinflammatory processes associated with AD progression. Also, it has been found an upregulation of heat shock proteins [35], and, as these proteins play essential roles in cellular proteostasis and stress response, they may potentially mitigate protein misfolding and aggregation, including Aβ peptides [122]. The findings from PD models revealed promising outcomes, although it is important to note that only two studies were included. One study [45] demonstrated significant reductions in oxidative stress markers, MDA, NO, GHS levels in the midbrain and striatum following PBM at 830 nm. In contrast, [44] reported no significant impact on cell survival or cortical cell density with PBM at 808 nm. However, they noted noteworthy neuroprotective effects against PD-related pathology, including nigral dopaminergic degeneration and striatal fiber denervation, particularly at higher fluences.

Moreover, it has been found a restoration of synaptic plasticity [35, 37], suggesting a potential mechanism underlying the observed improvements in cognitive function. PBM increased the expression GAP-43 and SYP to levels comparable to younger counterparts, indicating a potential role in promoting neuronal plasticity and synaptic function [33]. This restoration of synaptic plasticity is a crucial aspect of AD pathogenesis, as synaptic dysfunction is closely linked to cognitive decline in the disease [123], even at early stages [124]. This finding may be corroborated with the study of [37], which observed increased c-fos protein expression following the treatment [37]. In PD, the restoration of neurotransmitter levels, such as serotonin and norepinephrine, suggests a multifaceted neuroprotective effect [45].

### Limitations

In this systematic review, it is possible to outline some limitations. The heterogeneity of parameters and methodologies underscores the complexity of PBM research. It limits the establishment of causal relationships and highlights the importance of robust quality assessment measures, such as meta-analysis. In this review, there is a lack of quantitative assessment of the included articles. Also, some articles lack methodological details regarding PBM parameters, effect size is not commonly reported, and only few preclinical studies were longitudinal. These issues need to be addressed to overcome limitations in intervention protocols and ensure reproducibility. This will ensure the reliability and validity of findings in PBM related to aging, leading to an enhancement of the translational potential of PBM therapy.

## Conclusions

The articles included in this review describe a wide range of PBM methodologies, some resulting in benefits at a brain and behavioral level. Most clinical and experimental studies use wavelengths of 800, 810, or 1064 nm, but shorter wavelengths, such as 660 nm, or longer wavelengths, such as 1072 nm, have also been used. Other parameters such as type of wavelength (pulsed or continuous), frequency, intensity, irradiance, and days of application lack homogeneity and are sometimes unreported, preventing the replication of research. Female sex underrepresentation occurs in animal studies. The findings from both animal and human studies suggest that PBM holds promise as a non-invasive intervention for enhancing cognitive function. The observed improvements in memory, attention, and executive function indicate the potential therapeutic utility of PBM in mitigating cognitive decline associated with aging and neurodegenerative diseases. The efficacy of PBM in treating anxiety remains inconclusive, with limited evidence suggesting potential benefits in both animal and human studies. Further research, particularly with regards to anxiety-related disorders and locomotor activity, is warranted to elucidate the therapeutic potential of PBM and its safety profile. In terms of brain modulation, the decline in HbO post-PBM suggests enhanced brain functional organization, potentially boosting information processing efficiency and cognitive outcomes during working memory tasks in older adults. It suggests mitochondrial and hemodynamic effects. Also, cerebral perfusion and connectivity enhancements have been found, although scarce literature is available. Animal studies predominantly examined brain-related modifications, revealing promising effects of PBM in reversing metabolic alterations and enhancing mitochondrial function, as evidenced by restored CCO activity and ATP levels. Additionally, PBM demonstrated neuroprotective, anti-inflammatory, and immunomodulatory effects by attenuating oxidative stress, modulating apoptotic pathways, and reducing inflammatory cytokines while enhancing anti-inflammatory markers. Notably, PBM exerted significant impacts on intracellular signaling proteins, up-regulating survival, and neurotrophic factors while down-regulating pro-apoptotic markers. Moreover, PBM exhibited beneficial effects on synaptic plasticity, neurotransmitter levels, and cholinergic neurotransmission, highlighting its potential for promoting neuronal function and cognitive enhancement. Likewise, findings highlight the multifaceted neuroprotective effects of PBM therapy in AD models, encompassing not only Aβ plaque reduction but also improvements in synaptic plasticity, neuronal activity, and protein homeostasis. Discrepancies in study methodologies and parameters underscore the imperative for standardization and optimization of PBM treatment. These aspects are essential for a better analysis of the effects of PBM in aging and to avoid bias in the results. It remains a need for comprehensive assessment of its efficacy, particularly in long-term human cohorts.
